# Practical Diagnossi

**Published:** 1899-02

**Authors:** 


Practical Diagnossi. The Use of Symptoms in the Diagnosis of Disease. By Hobart Amory Hare, M. D., Professor of Therapeutics and Materia Medica in the Jefferson College of Philadel-Phia. Third edition, enlarged and thoroughly revised. In one octavo volume of 615 pages, with 204 engravings and 13 full-page colored plates. Clöth, $4.75, net. Lea Brothers & Co., Publishers, Philadelphia and New York.

We believe this book comes nearer meeting the demands of the profession than anything we have yet seen on the subject of physical diagnosis. The author's arrangement af both reading matter and index is directly the reverse of that adopted by other writers on this subject, and adds much to its value as a book of reference. His twelve years experience as teacher and practitioner has enabled him to treat the subject in a manner strikingly original and simple.

Every member and organ of the body is taken up separately, and the diagnostic value of abnormalities plainly given.

About one-fourth of the book is devoted to "Manifestations of Disease by Symptoms." This division is subdivided into nine chapters, with the following titles: "Chills, Fever and Subnormal Temperatures;" "Headache and Vertigo;" "Coma or unconsciousness;" "Convulsions or General Spasms;" "Vomiting;" "Cough and Expectoration;" "Pain;" "Tendon—Reflex and Muscle—Tone;" "Speech."

The book is complete, yet concisely written. It should be a part of every physician's library W. A. H.

